# Effect of angiography timing on acute kidney injury after cardiac surgery in patients with preoperative renal dysfunction

**DOI:** 10.1186/s12882-023-03144-y

**Published:** 2023-04-12

**Authors:** Wuhua Jiang, Qiwen Xie, Jiachang Hu, Xialian Xu, Jie Teng, Zhe Luo, Xiaoqiang Ding, Jiarui Xu

**Affiliations:** 1 Department of Nephrology, Zhongshan Hospital (Xiamen), Fudan University, Fujian, China; 2 Shanghai Institute of Kidney and Dialysis, Shanghai, China; 3 Department of Nephrology, Zhongshan Hospital, Fudan University, No 180 Fenglin Rd, Shanghai, China; 4 Department of Cardiac Surgery Intensive Care Unit, Zhongshan Hospital (Xiamen), Fudan University, Fujian, China

**Keywords:** Cardiac surgery, Acute kidney injury, Angiography

## Abstract

**Background:**

Cardiac surgery-associated acute kidney injury (AKI) is one of the common complications of cardiac surgery. Preoperative angiography helps assess heart disease but may increase the risk of AKI. Although more and more patients with preoperative renal dysfunction can undergo cardiac surgery with the advances in surgical techniques, there is little research on the effect of angiography on postoperative AKI in these patients. This study investigates whether angiography increases the risk of AKI after cardiac surgery in patients with preoperative renal dysfunction (15 ≤ eGFR < 60 ml/min/1.73m^2^).

**Methods:**

Patients with preoperative renal dysfunction (15 ≤ eGFR < 60 ml/min/1.73m^2^) who underwent angiography and cardiac surgery successively from January 2015 to December 2020 were retrospectively enrolled in this study. The primary outcome was postoperative AKI, defined as the Kidney Disease: Improving Global Outcomes Definition and Staging (KDIGO) criteria. Univariate analysis and multivariate regression were performed to identify the association between angiography timing and AKI.

**Results:**

A total of 888 consecutive eligible patients with preoperative renal dysfunction (15 ≤ eGFR < 60 ml/min/1.73m^2^) were enrolled in this study. The incidence of AKI was 48.31%. Male (OR = 1.903), the interval between angiography and surgery (0-2d OR = 2.161; 3-6d OR = 3.291), cross-clamp duration (OR = 1.009), were identified as predictors for AKI. The interval between angiography and surgery was also associated with AKI in the patients with 15 ≤ eGFR < 30ml/min/1.73m^2^ (0-2d OR = 4.826; 3-6d OR = 5.252), 30 ≤ eGFR < 45 ml/min/1.73m^2^ (0-2d OR = 2.952; 3-6d OR = 3.677), but not associated with AKI in patients with 45 ≤ eGFR < 60 ml/min/1.73m^2^.

**Conclusions:**

In patients with preoperative renal dysfunction, the interval between angiography and cardiac surgery (0-2d and 3-6d) was associated with AKI. For patients with poorer preoperative renal function, the interval between angiography and cardiac surgery is of great concern.

## Introduction

Cardiac surgery-associated acute kidney injury (AKI) is a serious and common complication after cardiac surgery. AKI is associated with increased mortality and end-stage renal disease, which may require ongoing dialysis and kidney replacement [1, 2]. With the rapid development of technology for cardiac surgery, the number of cardiac surgical procedures in China has significantly increased. Increasing numbers of patients with renal insufficiency are eligible for cardiac surgery, despite preoperative renal dysfunction being a risk factor for outcomes in cardiac surgery. The molecular mechanisms of AKI remain poorly understood, and no effective therapeutic strategies to target AKI are available [3]. Efforts toward the prevention, early diagnosis, and early treatment of AKI have therefore drawn extensive attention, especially for patients with preoperative renal dysfunction.

Coronary angiography is performed before heart surgery to determine the severity of coronary disease. Despite recent advancements in angiography techniques, contrast media’s harmful effects on kidney function raise major concerns. In recent years, some studies have investigated the effect of the time interval from coronary angiography to surgery on postoperative AKI, but the results are still controversial [4–7]. Especially in patients with preoperatively existing renal dysfunction, the effect of coronary angiography on postoperative AKI was rarely investigated. In our previous study [6], eGFR < 60ml/min/1.73m^2^ and angiography interval ≤ 7 days were independent risk factors for postoperative AKI. Therefore, this study aimed to investigate the effect of the time interval between coronary angiography and cardiac surgery on postoperative AKI in patients with preoperative renal dysfunction. The hypothesis that will be tested is (1) the shorter time interval between coronary angiography and surgery was associated with increased postoperative AKI, (2) the association became more significant as preoperative renal impairment worsened.

## Methods

### Patients and inclusion/exclusion criteria

Adult patients with preoperative renal dysfunction (15 ≤ eGFR < 60 ml/min/1.73m^2^) who underwent cardiac angiography plus valve/coronary artery bypass surgery or combined surgery in our hospital from January 2015 to December 2020 were included in this study. Exclusion criteria:(1) previous history of renal replacement therapy or kidney transplantation; (2) Preoperative AKI defined according to KDIGO criteria (preexisting increase in serum creatinine, decreased urine output, or both); (3) Incomplete medical history data; (4) Patients who died within 48 h after ICU admission; (5) Patients underwent urgent or emergent surgery. The Institutional Ethics Committee of Zhongshan Hospital approved the study design and data collection, and informed consent was waived due to the retrospective retrieval of the patient’s data.

### Design

This study was a retrospective observational study. Clinical data were obtained from electronic medical records, including demographic characteristics, comorbidities, baseline laboratory data, type of surgery, duration of cardio-pulmonary bypass (CPB), surgery bleeding (classified with Bleeding Academic Research Consortium grades), postoperative medication, urine output, and prognosis, including length of hospital stay and in-hospital mortality. Estimates of glomerular filtration rate (eGFR) were calculated using the CKD-EPI formula based on patient characteristics and baseline serum creatinine (SCr), which was defined as the latest preoperative measurement. SCr was measured at least once a day during the postoperative ICU as a standard of care. Renal function tests were performed during the first three days after a return to the ward from the ICU. If the patient’s condition is stable, the blood test will be taken on alternate days from the fourth day and continue until discharge. The amount of intake and output volume was recorded daily.

All patients in the dataset received isotonic contrast media with an iodine concentration of 320 mg/mL (iodixanol, Visipaque®, GE Healthcare, Ireland). Known nephrotoxic drugs were discontinued 24 h before angiography. Besides, adequate hydration of intravenous normal saline before angiography (1 ml/kg·h for 12 h), limiting contrast media volume and intravenous sodium bicarbonate after angiography were routinely executed as standard protocol. The time interval between the angiography and the surgery was defined (days). Angiography on the day of surgery was coded as interval 0. To clarify the exact relationship and threshold between the interval between angiography and surgery and the incidence of AKI, we included the time threshold summarized in the existing literature: 3 days and 7 days. The two thresholds were used to classify patients into three classes (0-2d, 3-6d, 7days and above), and the interval was converted from a continuous variable to a categorical data for regression analysis. Since we hypothesize that the shorter interval is associated with AKI, we set the interval ≥ 7days as a reference indicator.The dose of contrast medium was defined (ml). To more specifically reflect the effect of contrast volume in patients with renal dysfunction, we used patients’ eGFR to adjust contrast volume (contrast volume/eGFR). The data of access site was collected as well.

**Fig. 1:**
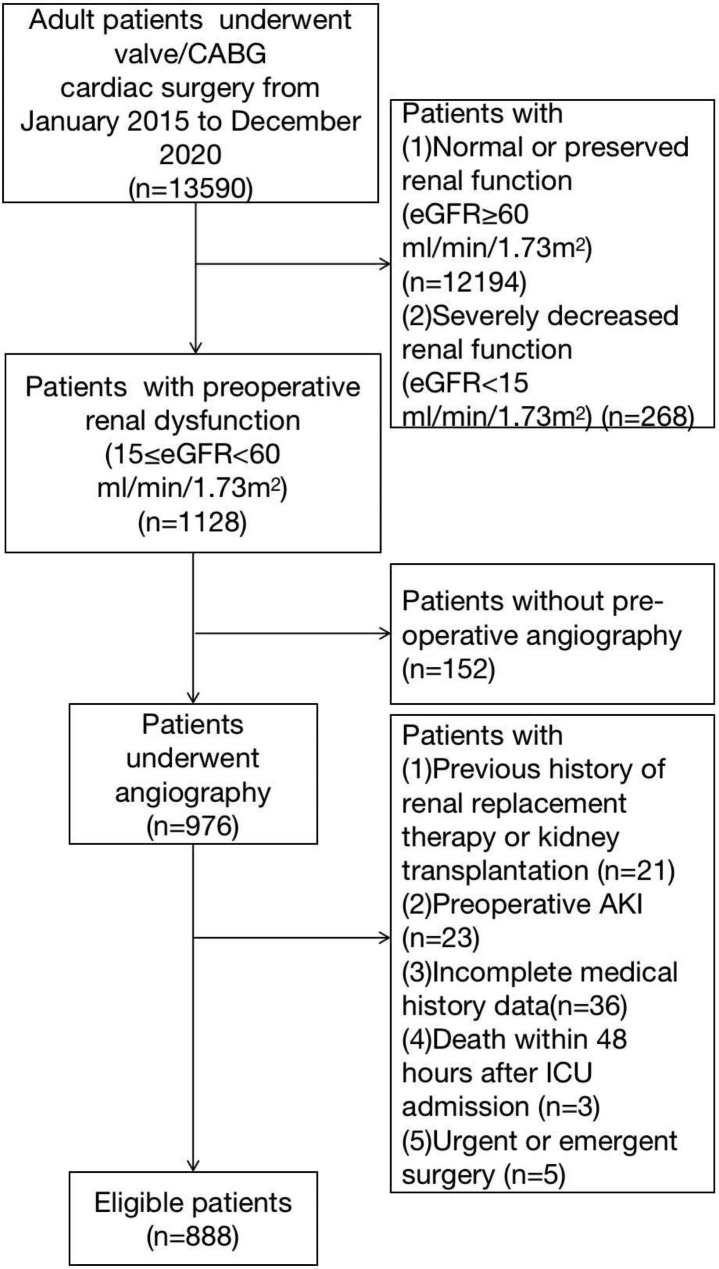
The flow chart of patient enrollment

Whether or not patients underwent cardiac surgery on the day of angiography, a blood test including blood routine and renal function test will be performed the next morning after the angiography. If the patient has previous renal function results, we compare and determine whether the patient has prexsisting AKI before surgery. These patients with prexsisting AKI were excluded from the study (Fig. 1). All data were followed up until discharge or death.

The primary outcome was postoperative AKI. Patients were divided into non-AKI group and AKI group according to the occurrence of AKI, defined according to KDIGO guidelines [8]. The risk factors of postoperative AKI were investigated.

To observe and compare the association between angiography and postoperative AKI in patients with different degrees of preoperative eGFR, patients were divided into three subgroups based on preoperative eGFR (group A:15 ≤ eGFR < 30ml/min/1.73m^2^, group B:30 ≤ eGFR < 45,ml/min/1.73m^2^ group C:45 ≤ eGFR < 60 ml/min/1.73m^2^). On the one hand, this grouping method is conducive to the detailed comparison of clinical characteristics of patients with different basic renal function; on the other hand, it draws reference from the stages 3a, 3b and 4 of chronic kidney disease, which is in line with the conventional understanding of scholars on the disease.

All statistical analyses were performed using the SPSS 25.0 statistical package (IBM, Armonk, NY, USA). They were expressed as mean ± standard deviation for normally distributed data, median and interquartile range for continuous variables that are not normally distributed, and number (%) for categorical variables. The Kolmogorov-Smirnov test was used to check the normality and homogeneity of variance of all data. One-way Student t-test was used to determine the difference between groups for P values and the nonparametric test for other data. Fisher exact test or chi-square test was used to compare changes in categorical variables. Univariate logistic regression analysis was used to compare patient characteristics, intraoperative variables and postoperative variables to determine the risk factors of postoperative AKI. Odds ratios (OR) of AKI predictors were calculated with 95% confidence intervals (CI). The multivariable logistic regression analysis was further performed using the stepwise forward selection for the variables with a P value of < 0.05 considered predictive for postoperative AKI. If the interval between angiography and surgery was associated with the onset of AKI, we would screen the available literature for thresholds to identify specific time windows of this risk factor. Of all the comparisons, P < 0.05 was considered statistically significant.

## Results

### Basic characteristics

The flow chart of patient enrollment was shown in Fig. 1. A total of 888 patients with preoperative kidney dysfunction were included in this study whereas 48.31% (n = 429) developed AKI after surgery. Mortality was significantly higher in patients with AKI than in non-AKI (3.7 vs. 0.7%, P = 0.002). The length of hospital stay was significantly longer [14 (11,17) vs. 15 (12,21) days, P = 0.005]. The proportion of males and hypertension in the AKI group was significantly higher than in the non-AKI group. In laboratory test data, compared with the non-AKI group, the AKI group had lower preoperative hemoglobin and albumin. Among the included procedures, the incidence of AKI was higher in the combined surgery group. In addition, the AKI group had a longer aortic clamping time (Table 1).

**Table 1:** Perioperative Characteristics of the Study Population

Characteristics		Non-AKI (N = 459)	Postoperative AKI (N = 429)		*p*
*Demographic data*					
Male (%)		242 (52.7)	265 (61.8)		0.007
Age (years)		66.04 ± 7.89	66.48 ± 7.62		0.402
BMI (kg/m^2^)		23.43 ± 2.96	23.47 ± 3.07		0.858
*Comorbidities*					
Hypertension (%)		241 (52.6)	256 (59.7)		0.036
DM (%)		82 (17.9)	85 (19.8)		0.492
NYHA grade 3–4 (%)		289 (67.8)	282 (68.8)		0.824
LVEF (%)		58 ± 9	56 ± 10		0.029
*Angiography data*					
Interval between angiography and surgery (days)		4 (2,7)	4 (2,6)		0.041
0-2d		187 (40.7)	196 (45.7)		0.154
3-6d		136 (29.6)	144 (33.6)		0.220
≥7d		136 (29.6)	89 (20.7)		0.003
Contrast volume (ml)		70 (60,90)	70 (60,90)		0.432
Contrast volume/ eGFR ratio		1.72 ± 1.49	1.65 ± 0.81		0.374
Femoral artery approach (%)		56 (12.2)	47 (11.0)		0.601
*Baseline laboratory indices*					
Hemoglobin (g/L)		129.78 ± 16.03	126.01 ± 18.12		0.001
Albumin (g/L)		40.15 ± 3.60	39.31 ± 3.70		0.001
BUN (mmol/L)		9.53 ± 4.82	9.49 ± 3.77		0.907
Serum creatinine (µmol/L)		128.32 ± 64.47	129.98 ± 37.31		0.641
eGFR (ml/min/1.73m^2^)		49.35 ± 10.28	48.16 ± 9.46		0.074
15 ≤ eGFR < 30 ml/min/1.73m^2^		25 (5.5)	23 (5.4)		0.997
30 ≤ eGFR < 45 ml/min/1.73m^2^		81 (19.38)	103 (24.0)		0.02
45 ≤ eGFR < 60 ml/min/1.73m^2^		353(76.9)	303 (70.6)		0.039
Uric acid(µmol/L)		465.89 ± 177.94	486.22 ± 163.35		0.079
*Surgery*					
Sole Valve (%)		276 (60.1)	241 (56.2)		0.336
Sole CABG (%)		163 (35.5)	140 (32.6)		0.725
Valve & CABG (%)		20 (4.4)	48 (11.2)		0.001
CPB duration (mins)		90 (71,115)	104 (76,129)		0.001
Cross-clamp duration (mins)		57.34 ± 24.89	61.99 ± 27.08		0.036
BARC type 3–4 (%)		132 (28.8)	143 (33.3)		0.147
*Prognosis*					
In-hospital mortality		3 (0.7)	16 (3.7)		0.002
Length of ICU stay (hours)		42 (21,70)	65 (26,113)		0.001
Length of hospital stay (days)		14 (11,17)	15 (12,21)		0.005

AKI: Acute kidney injury; BARC: Bleeding Academic Research Consortium; BMI: Body Mass Index; BUN: Blood Urea Nitrogen; CABG: Coronary artery bypass grafting; CPB: Cardiopulmonary bypass; DM: Diabetes mellitus; eGFR: Estimated glomerular filtration rate, calculated by CKD-EPI formulae; ICU: intensive care unit; LVEF: Left ventricular ejection fraction; NYHA: New York Heart Association

The values are expressed as the median (IQR) and mean ± SD or number (%)

P-values are the results of unpaired t-test or Kolmogorov-Smirnov test for continuous variables, and χ2 test or Fisher’s exact test for categorical variables

Univariate logistic regression analysis showed that male, preoperative hypertension, increased left ventricular ejection fraction, the interval between angiography and surgery (0-2d, 3-6d), combined surgery, prolonged cross-clamp duration, elevated preoperative hemoglobin and albumin were associated with postoperative AKI (Table 2). The above factors were further included in the multivariate logistic regression, and the results showed that male (OR = 1.903), the interval between angiography and surgery (0-2d OR = 2.161; 3-6d OR = 3.291),and cross-clamp duration (OR = 1.009) were independent risk factors for postoperative AKI (Table 2).

**Table 2:** Logistic regression of risk factors for AonC.

	Univariate analysis				Multivariate analysis		
	OR	95%CI	P value		OR	95%CI	P value
Male	1.449	1.109–1.893	0.007		1.903	1.213–2.984	0.005
Age	1.007	0.990–1.025	0.402				
BMI	1.004	0.960–1.050	0.857				
Interval^†^	0.928	0.899–0.957	0.001		0.931	0.873–0.993	0.03
0-2d	1.602	1.147–2.237	0.008		2.161	1.094–4.270	0.026
3-6d	1.618	1.134–2.309	0.006		3.291	1.622–6.677	0.001
≥7d	Reference				Reference		
Contrast volume	0.997	0.992–1.003	0.305				
Contrast volume eGFR ratio	0.949	0.844–1.068	0.384				
Hypertension	1.332	1.021–1.739	0.035		1.232	0.769–1.972	0.489
DM	1.133	0.809–1.587	0.467				
NYHA grade 3–4	1.044	0.780–1.398	0.770				
LVEF	0.982	0.966–0.998	0.03		0.980	0.956–1.005	0.052
Hemoglobin	0.987	0.979–0.995	0.001		0.986	0.972–1.001	0.056
Albumin	0.938	0.903–0.976	0.001		0.919	0.869–1.023	0.199
BUN	0.998	0.967–1.030	0.907				
Serum creatinine	1.001	0.998–1.003	0.642				
eGFR	0.988	0.975–1.001	0.075				
15 ≤ eGFR < 30 ml/min/1.73m2	1.048	0.582–1.884	0.877				0.536
30 ≤ eGFR < 45 ml/min/1.73m2	1.448	1.041–2.013	0.028				0.053
45 ≤ eGFR < 60 ml/min/1.73m2	Reference				Reference		
Uric acid	1.001	1.000-1.002	0.088				
CPB duration	1.003	1.000-1.007	0.087				
Cross-clamp duration	1.007	1.00-1.013	0.039		1.009	1.000-1.017	0.040
Combined surgery	2.749	1.587–4.762	< 0.001		2.882	0.865–6.776	0.346

AonC: Acute kidney injury superimposed on chronic kidney disease; BMI: Body Mass Index; BUN: Blood Urea Nitrogen; CABG: Coronary artery bypass grafting; CPB: Cardiopulmonary bypass; DM: Diabetes mellitus; eGFR: Estimated glomerular filtration rate, calculated by CKD-EPI formulae; LVEF: Left ventricular ejection fraction; NYHA: New York Heart Association, NS: not significant

† Interval refers to the time between contrast exposure and cardiac surgery

### Subgroup analysis

Patients were classified according to preoperative renal function (A:15 ≤ eGFR < 30ml/min/1.73m^2^, B:30 ≤ eGFR < 45ml/min/1.73m^2^, C:45 ≤ eGFR < 60 ml/min/1.73m^2^) (Table 3), we also included the interval threshold reported in the existing literature. Logistic regression performed in each subgroup (Table 4) showed that in the subgroup of group A (15 ≤ eGFR < 30 ml/min/1.73m^2^) and group B(30 ≤ eGFR < 45 ml/min/1.73m^2^), the interval between angiography and surgery was associated with AKI in these patients [15 ≤ eGFR < 30:(0-2d OR = 4.826; 3-6d OR = 5.252); 30 ≤ eGFR < 45 ml/min/1.73m^2^:(0-2d OR = 2.952; 3-6d OR = 3.677)]. In the subgroup of 45 ≤ eGFR < 60ml/min/1.73m^2^, the interval between angiography and surgery was not statistically significantly associated with AKI.

**Table 3:** Perioperative Characteristics of the Subgroups Classified with Baseline eGFR

	15 ≤ eGFR < 30 ml/min/1.73m^2^			30 ≤ eGFR < 45 ml/min/1.73m^2^			45 ≤ eGFR < 60 ml/min/1.73m^2^		
Characteristics	Non-AKI (N = 25)	Postoperative AKI (N = 23)	*p*	Non-AKI (N = 81)	Postoperative AKI (N = 103)	*p*	Non-AKI (N = 353)	Postoperative AKI (N = 303)	*p*
*Demographic data*									
Male (%)	12 (48)	8 (34.8)	0.394	36 (44.4)	77 (74.8)	< 0.001	193 (54.8)	180 (59.4)	0.269
Age (years)	67.68 ± 5.88	65.43 ± 8.77	0.398	66.63 ± 8.71	66.50 ± 7.56	0.914	65.83 ± 7.89	66.6 ± 7.58	0.209
BMI (kg/m^2^)	22.99 ± 2.61	22.97 ± 2.91	0.981	23.29 ± 3.00	23.19 ± 3.10	0.825	23.48 ± 2.97	23.60 ± 3.06	0.62
*Comorbidities*									
Hypertension (%)	16 (64)	19 (82.6)	0.200	47 (58.0)	59 (57.3)	0.882	177 (50.4)	178 (58.7)	0.034
DM (%)	7 (28)	11 (47.8)	0.234	22 (27.2)	23 (22.3)	0.491	52 (14.8)	51 (16.8)	0.519
NYHA grade 3–4 (%)	15 (68.2)	17 (77.3)	0.736	54 (70.1)	72 (73.5)	0.735	219 (67.2)	193 (66.6)	0.913
LVEF	54 ± 9	53 ± 11	0.812	59 ± 10	56 ± 12	0.151	58 ± 59	57 ± 9	0.152
Interval between angiography and surgery (days)	7 (3,17)	3 (1,6)	0.014	6 (3,12)	5 (2,7)	0.015	4 (2,6)	4 (2,6)	0.786
0-2d	7 (28.0)	13 (56.5)	0.078	25 (30.9)	41 (39.8)	0.220	155 (43.9)	142 (46.9)	0.479
3-6d	4 (16.0)	7 (30.4)	0.311	22 (27.2)	38 (36.9)	0.205	110 (31.3)	99 (32.7)	0.737
≥7d	14 (56)	3 (13.0)	0.003	34 (42.0)	24 (23.3)	0.01	88 (25.0)	62 (20.5)	0.192
Contrast volume (ml)	70 (50,90)	65 (50,100)	0.943	70 (60,90)	70 (60,90)	0.914	70 (60,90)	70 (60,90)	0.449
Contrast volume/ eGFR ratio	5.09 ± 4.81	3.42 ± 1.88	0.126	2.04 ± 0.95	1.97 ± 0.707	0.601	1.41 ± 0.425	1.41 ± 0.435	0.894
*Baseline laboratory indices*									
Hemoglobin (g/L)	114.56 ± 14.65	107.41 ± 19.24	0.156	130.08 ± 14.91	126.93 ± 18.08	0.216	131.17 ± 15.87	127.07 ± 17.31	0.002
Albumin (g/L)	39.0 ± 3.28	37.79 ± 6.09	0.406	39.97 ± 3.99	39.30 ± 3.49	0.254	40.32 ± 3.55	39.4 ± 3.54	0.002
BUN (mmol/L)	14.55 ± 4.65	19.69 ± 10.06	0.035	11.78 ± 4.84	11.54 ± 4.25	0.729	8.33 ± 2.68	8.38 ± 2.80	0.807
Serum creatinine (µmol/L)	228.23 ± 66.76	312.48 ± 182.23	0.042	145.19 ± 22.454	154.41 ± 22.92	0.007	111.58 ± 16.39	114.14 ± 16.86	0.05
Uric acid(µmol/L)	519.25 ± 136.65	722.26 ± 514.89	0.113	502.67 ± 139.46	545.94 ± 122.76	0.04	443.19 ± 123.97	464.99 ± 171.19	0.073
*Surgery*									
Sole Valve (%)	15 (60)	14 (60.9)	0.752	42 (51.9)	53 (51.5)	0.898	218 (61.9)	174 (57.4)	0.132
Sole CABG (%)	9 (36)	8 (34.7)	0.662	36 (44.4)	38 (36.9)	0.152	118 (33.6)	94(31.0)	0.685
Valve & CABG (%)	1 (4.0)	1 (4.3)	0.886	3 (3.7)	12 (11.7)	0.069	16 (4.5)	35 (11.5)	0.003
CPB duration (mins)	110 (82,121)	97 (82,119)	0.611	105 (84,136)	101 (76,132)	0.574	85 (68,112)	105(77,129)	< 0.001
Cross-clamp duration (mins)	65.94 ± 27.48	59 ± 20.9	0.482	61.90 ± 27.01	61.61 ± 29.65	0.960	55.86 ± 24.34	62.27 ± 26.67	0.011
*Prognosis*									
In-hospital mortality	1 (4)	2 (8.7)	0.601	0 (0)	6(5.8)	0.019	2 (0.6)	8 (2.6)	0.031
Length of hospital stay (days)	19 (13,33)	18 (12,24)	0.347	15 (11,22)	15 (12,21)	0.652	13 (11,17)	15 (12,20)	0.003

AKI: Acute kidney injury; BMI: Body Mass Index; BUN: Blood Urea Nitrogen; CABG: Coronary artery bypass grafting; CPB: Cardiopulmonary bypass; DM: Diabetes mellitus; eGFR: Estimated glomerular filtration rate, calculated by CKD-EPI formulae; ICU: intensive care unit; LVEF: Left ventricular ejection fraction; NYHA: New York Heart Association

The values are expressed as the median (IQR) and mean ± SD or number (%)

P-values are the results of unpaired t-test or Mann–Whitney U test for continuous variables, and χ2 test or Fisher’s exact test for categorical variables

**Table 4:** Logistic regression of risk factors for AonC in Subgroups

	Univariate analysis				Multivariate analysis		
	OR	95%CI	P value		OR	95%CI	P value
15 ≤ eGFR < 30 ml/min/1.73m^2^							
†Interval	0.863	0.751–0.992	0.038		0.813	0.671–0.986	0.035
0-2d					4.826	2.274–7.279	0.004
3-6d					5.252	1.788–9.796	0.011
≥7d	Reference				Reference		
BUN	1.101	1.002–1.189	0.045		1.004	0.967–1.102	0.249
Serum creatinine	1.006	1.000-1.013	0.072		1.003	0.997–1.012	0.094
30 ≤ eGFR < 45 ml/min/1.73m^2^							
Male	3.703	1.983–6.911	< 0.001		3.795	1.872–7.695	0.001
†Interval	0.893	0.831–0.958	0.002		0.892	0.822–0.969	0.007
0-2d					2.952	1.289–6.762	0.01
3-6d					3.677	1.566–8.635	0.003
≥7d	Reference				Reference		
Serum creatinine	1.018	1.004–1.032	0.008		1.008	0.995–1.023	0.765
Uric acid	1.003	1.000-1.005	0.042		1.001	0.996–1.003	0.153
45 ≤ eGFR < 60 ml/min/1.73m^2^							
Hypertension	1.438	1.052–1.964	0.023		1.674	1.110–2.523	0.014
Hemoglobin	0.986	0.977–0.996	0.006		0.984	0.970–1.005	0.068
Albumin	0.936	0.893–0.981	0.006		0.918	0.905–1.004	0.165
Serum creatinine	1.011	1.002–1.021	0.022		1.003	1.007–1.033	0.003
Combined surgery	2.741	1.468–5.116	0.002		2.745	0.965–5.245	0.238
CPB duration	1.005	1.000-1.010	0.048		1.004	0.995–1.011	0.309
Cross-clamp duration	1.010	1.003–1.018	0.008		1.012	1.004–1.021	0.004

AonC: Acute kidney injury superimposed on chronic kidney disease; BUN: Blood urea nitrogen; CPB: Cardiopulmonary bypass; eGFR: Estimated glomerular filtration rate, calculated by CKD-EPI formulae;OR: odds ration; CI: confidence interval

† Interval refers to the time between contrast exposure and cardiac surgery

## Discussion

In this retrospective study, we revealed the adverse effect of the interval between contrast surgery and cardiac surgery on postoperative AKI in patients with advanced severity of preoperative renal dysfunction. To clarify the relationship between specific interval time and AKI, we adopted and analyzed interval (3 days and 7 days) reported in previous literature and found that the interval between angiography and surgery (0-2d and 3-6d) was associated with the increased risk of AKI. And the association was more significant in patients with severe preoperative renal dysfunction 15 ≤ eGFR < 45ml/min/1.73m^2^.

With the development of cardiac surgery technology, an increasing number of patients with preoperative renal dysfunction have been able to undergo cardiac surgery, and the development of AKI in these patients will seriously affect their kidney and overall prognosis [9, 10]. Thus, early identification of risk factors is of great significance for the prognosis of patients with preoperative renal dysfunction. Contrast media is an important cause of hospital-acquired AKI. Adequate hydration, hypo- or iso-osmolar iodine contrast media are currently in clinical use. Although hydration is an important method for the prevention of contrast induced nephropathy, it should be applied cautiously in patients with renal dysfunction. Once volume overload or even heart failure occurs, it is not conducive for patients to prepare for cardiac surgery. On the other hand, cardiac surgery performed when the kidney has not yet recovered from contrast media nephrotoxicity can increase the risk of AKI, seriously affecting the kidney and overall prognosis. Therefore, this study focused on patients with preoperative renal dysfunction and found that the interval between angiography and surgery (0-2d and 3-6d) was associated with the onset of AKI.

The American College of Cardiology/American Heart Association guidelines for managing patients with valve heart disease has a class I level of recommendation to perform angiography in patients scheduled for valve surgery [11]. Patients with renal dysfunction are often complicated with cardiovascular diseases such as vascular calcification. Therefore, preoperative angiographic examination and tolerance assessment are of significance. As rare modifiable risk factors of postoperative AKI, the interval between angiography and surgery, along with the access site and contrast media volume, have drawn more and more attention.

Previous studies [4, 5, 12, 13] mainly focused on the effect of the interval between angiography and surgery on postoperative AKI in the population which mainly consists of patients with sufficient renal function. To date, there has been little agreement on the concrete relationship between interval and AKI, due to different surgical approaches and definitions of AKI among studies. Given that the majority of the population included in previous studies were patients with normal renal function, we hypothesized that the association between a shorter time interval and the onset of AKI was significant in patients with severely impaired pre-operative renal function.

Compared with previous studies, the incidence of AKI after cardiac surgery in the present study is higher than that reported in the previous studies. The reason is that the population in our study had a worse basic renal function, was susceptible to insult, and was prone to AKI. To elucidate the explicit association between preoperative eGFR angiography and postoperative AKI in patients with different degrees of eGFR, we performed the subgroup investigation. After classifying patients according to different preoperative renal functions, we found that in patients with 30 ≤ eGFR < 45 ml/min/1.73m^2^, 15 ≤ eGFR < 30ml/min/1.73m^2^, the operative interval less than 7 days (including 0-2days and 3-6days) was associated with postoperative AKI, but we did not find a similar association in patients with 45 ≤ eGFR < 60ml/min/1.73m^2^. In this subgroup, there was no significant difference in the interval between AKI and non-AKI patients (Table 3). These results suggested that the association between interval and AKI is stronger in patients with severe renal dysfuntion. In patients with mild renal dysfuntion (45 ≤ eGFR < 60 ml/min/1.73m^2^), the relationship was ‘weaker’ than conventional risk factors (hypertension, increased preoperative creatinine, etc.). In previous studies, patients with normal or preserved renal function were mainly enrolled, while patients with severe renal dysfuntion were few. These previous studies failed to reach a consistent conclusion on the relationship between interval and AKI. Whether the interval affects AKI in patients with different severity of renal dysfunction needs to be elucidated by prospective intervention study. Nevertheless, further prospective studies may include patients with severely impaired renal function (15 ≤ eGFR < 45ml/min/1.73m^2^) to determine whether prolonging surgical intervals over 7 days can reduce the incidence of AKI.

On the other hand, we found that in group C patients, conventional risk factors such as complex surgery, hemoglobin, albumin, and CPB duration were significantly different in univariate analysis. However, after multivariate analysis, these factors did not show statistical difference, possibly because they had weaker association with AKI than hypertension, serum creatinine and cross-clamp duration. However, in Table 2, which encompassed the entire cohort, hypertension and serum creatine were not statistically associated with AKI. Therefore, through subgroup analysis of patients, the risk factors of patients with different preoperative renal function can be more clearly identified. Because sweeping analyses can lead to elusive conclusions similar to previous studies.

It is also worth noting that in this study, as a continuous variable, interval is inversely associated with the onset of AKI, whether in the entire cohort or in group A and group B in the subgroup analysis, but we believe this is not of clinical significance. Therefore, we classified patients into three groups based on interval: 0-2d, 3–6, and ≥ 7d. As shown in Tables 1 and 3, the 0-2d and 3-6d proportion showed no significant difference between AKI and non-AKI groups, while the ≥ 7d proportion showed significant difference between AKI and non-AKI groups. However, when we set the ≥ 7d group as the reference indicator and included the time interval as the categorical variable in the multivariate regression analysis, the 0-2d class and 3-6d class showed statistically significant association with AKI. The reason, we believe, is that the interval ≥ 7d has a strong association with AKI, so that until when it was taken as the reference indicator, 0-2d and 3-6d were associated with AKI. However, this study was not an intervention study, and we were not able to intervene the interval. Therefore, whether the interval ≥ 7d can help reduce the incidence of AKI is elusive. Therefore we set it as the reference indicator to highlight the association between interval < 7d (0-2d & 3-6d) and postoperative AKI.

Another interesting finding was that a longer time interval (3-6d) seemed to be more detrimental compared to a shorter time interval (0-2d). To elaborate this finding, we compared all variables between the two groups (0-2d vs. 3-6d), including laboratory tests, and found that the proportion of males (62.1 vs. 51.2%, p = 0.006) and hypertension (59.6 vs. 48.0%, p = 0.004) were higher in the 3-6d group than in the other group 0-2d. As was shown in the multivariate regression results, AKI was associated with males in this study (OR = 1.903), while hypertension was only associated with AKI in univariate analysis. Therefore, we speculate that the potential reason for the more detrimental effect of the 3-6d group may be that the number of patients in the 3-6d group was less than that in the 0-2d group, while the proportion of male in the 3-6d group was higher than that in the 0-2d group, so the association with AKI was more prominent in the 3-6d group.

Although previous studies have shown that preoperative renal dysfunction is a risk factor for postoperative AKI, eGFR did not stand out in the present study. We think it may be due to the patients included in this study. Previous studies mainly included patients with normal renal function. Compared with patients with normal renal function, the amount of patients with reduced eGFR are small after all, but these patients have a higher incidence of AKI, so the association between AKI and eGFR can be evident. However, all the patients included in the present study were patients with reduced eGFR. Although we classified them by different eGFR, the proportion of AKI in each group was very high. Therefore, when we explore the risk factors associated with AKI in these patients, some “weaker” but clinically significant risk factors would appear. In contrast, some conventional risk factors may not stand out in this particular population.

Our study still has some shortcomings. Firstly, the present study is a single-center retrospective case-control study, and more prospective multicenter randomized controlled studies are yet needed to validate whether prolonged surgical intervals can reduce AKI. Secondly, we have found in previous studies that high-dose contrast media was associated with postoperative AKI. In this study, we analyzed eGFR-adjusted contrast media volume in order to more accurately reflect the adverse effects of contrast agent on patients with renal dysfuntion. However, there was no significant association between contrast media volume and AKI in this study, which needs to be further validated in future studies. Thirdly, although we followed up on the renal function of patients during hospitalization, the frequency of renal function tests between angiography and surgery differed. Some patients (n = 14) underwent surgery on the day of angiography, so it was impossible to distinguish whether postoperative AKI was caused by contrast medium toxicity or cardiac surgery. The etiology identification requires a kidney biopsy. We will discuss kidney biopsy’s feasibility in postoperative AKI patients with surgical departments. Fourthly, stable patients were given blood tests on alternate days from the fourth day of ICU departure until discharge. Although this frequency is reduced compared to that in the ICU, laboratory tests and urine output follow-up can meet the time threshold required to observe KDIGO’s definition of AKI. Renal function tests were performed during the first three days after a return to the ward from the ICU. But this was a retrospective study, and we were not able to intervene in patients’ blood testing plans, which is regrettable and we hope this defect could be refined in future prospective studies.

In conclusion, a shorter interval between angiography and surgery (0-2d and 3-6d) was associated with a increased risk of postoperative AKI in patients with preoperative renal dysfunction. In patients with severe preoperative renal dysfunction (15 ≤ eGFR < 45ml/min/1.73m^2^), the risk of AKI was increased if they undergo cardiac surgery shortly after coronary angiography. In patients with severe renal dysfunction, the interval between angiography and cardiac surgery is of great concern.

## Data Availability

The datasets used and/or analysed during the current study available from the corresponding author on reasonable request.

## Abbreviations

AKI: Acute kidney injury
BMI: Body Mass Index
BUN: Blood Urea Nitrogen
CABG: Coronary artery bypass grafting
CPB: Cardiopulmonary bypass
DM: Diabetes mellitus
eGFR: Estimated glomerular filtration rate, calculated by CKD-EPI formulae
ICU: intensive care unit
LVEF: Left ventricular ejection fraction
NYHA: New York Heart Association

## References

[1] Tseng PY, Chen YT, Wang CH, Chiu KM, Peng YS, Hsu SP, Chen KL, Yang CY, Lee OK (2020). Prediction of the development of acute kidney injury following cardiac surgery by machine learning. Crit Care.

[2] Hu J, Chen R, Liu S, Yu X, Zou J, Ding X (2016). Global incidence and outcomes of adult patients with acute kidney Injury after Cardiac surgery: a systematic review and Meta-analysis. J Cardiothorac Vasc Anesth.

[3] Wang Y, Bellomo R (2017). Cardiac surgery-associated acute kidney injury: risk factors, pathophysiology and treatment. Nat Rev Nephrol.

[4] Liu K, Li M, Li L, Wu B, Xu X, Ge Y, Mao H, Xing C (2021). The effect of coronary angiography timing on cardiac surgery Associated Acute kidney Injury incidence and prognosis. Front Med (Lausanne).

[5] Kim K, Joung KW, Ji SM, Kim JY, Lee EH, Chung CH, Choi IC (2016). The effect of coronary angiography timing and use of cardiopulmonary bypass on acute kidney injury after coronary artery bypass graft surgery. J Thorac Cardiovasc Surg.

[6] Jiang W, Yu J, Xu J, Shen B, Wang Y, Luo Z, Wang C, Ding X, Teng J (2018). Impact of cardiac catheterization timing and contrast media dose on acute kidney injury after cardiac surgery. BMC Cardiovasc Disord.

[7] Dayan V, Stanham R, Soca G, Genta F, Marino J, Lorenzo A (2017). Early surgery after angiography in patients scheduled for valve replacement. Asian Cardiovasc Thorac Ann.

[8] Kellum JA, Lameire N (2013). Diagnosis, evaluation, and management of acute kidney injury: a KDIGO summary (part 1). Crit Care.

[9] Palomba H, Castro I, Yu L, Burdmann EA (2017). The duration of acute kidney injury after cardiac surgery increases the risk of long-term chronic kidney disease. J Nephrol.

[10] Coca SG, Singanamala S, Parikh CR (2012). Chronic kidney disease after acute kidney injury: a systematic review and meta-analysis. Kidney Int.

[11] Nishimura RA, Otto CM, Bonow RO, Carabello BA, Erwin JP 3rd, Fleisher LA, Jneid H, Mack MJ, McLeod CJ, O’Gara PT, et al. 2017 AHA/ACC focused update of the 2014 AHA/ACC Guideline for the management of patients with Valvular Heart Disease: a report of the American College of Cardiology/American Heart Association Task Force on Clinical Practice Guidelines. J Am Coll Cardiol. 2017;70(2):252–89. 10.1016/j.jacc.2017.03.011 28315732

[12] Medalion B, Cohen H, Assali A, Vaknin Assa H, Farkash A, Snir E, Sharoni E, Biderman P, Milo G, Battler A (2010). The effect of cardiac angiography timing, contrast media dose, and preoperative renal function on acute renal failure after coronary artery bypass grafting. J Thorac Cardiovasc Surg.

[13] Borde DP, Asegaonkar B, Apsingekar P, Khade S, Khodve B, Joshi S, George A, Pujari A, Deodhar A (2019). Influence of time interval between coronary angiography to off-pump coronary artery bypass surgery on incidence of cardiac surgery associated acute kidney injury. Indian J Anaesth.

